# The ADaptation and Anticipation Model (ADAM) of sensorimotor synchronization

**DOI:** 10.3389/fnhum.2013.00253

**Published:** 2013-06-10

**Authors:** M. C. (Marieke) van der Steen, Peter E. Keller

**Affiliations:** ^1^Max Planck Research Group “Music Cognition and Action”, Max Planck Institute for Human Cognitive and Brain SciencesLeipzig, Germany; ^2^Music Cognition and Action Group, The MARCS Institute, University of Western SydneySydney, NSW, Australia

**Keywords:** sensorimotor synchronization, computational model, temporal adaptation, error correction, temporal anticipation, predictive internal models

## Abstract

A constantly changing environment requires precise yet flexible timing of movements. Sensorimotor synchronization (SMS)—the temporal coordination of an action with events in a predictable external rhythm—is a fundamental human skill that contributes to optimal sensory-motor control in daily life. A large body of research related to SMS has focused on adaptive error correction mechanisms that support the synchronization of periodic movements (e.g., finger taps) with events in regular pacing sequences. The results of recent studies additionally highlight the importance of anticipatory mechanisms that support temporal prediction in the context of SMS with sequences that contain tempo changes. To investigate the role of adaptation and anticipatory mechanisms in SMS we introduce ADAM: an ADaptation and Anticipation Model. ADAM combines reactive error correction processes (adaptation) with predictive temporal extrapolation processes (anticipation) inspired by the computational neuroscience concept of internal models. The combination of simulations and experimental manipulations based on ADAM creates a novel and promising approach for exploring adaptation and anticipation in SMS. The current paper describes the conceptual basis and architecture of ADAM.

## Introduction

An intriguing question about motor control in daily life is how people effectively time their coordinated actions during everyday activities. The question is especially interesting when one considers that coordination needs to occur in a constantly changing environment and with other people who are also dynamic in their behavior. Precise but flexible motor timing is an important aspect of successful coordination. Sensorimotor synchronization (SMS) is a fundamental human skill that is the basis of numerous forms of behavioral coordination. Broadly speaking, SMS is the temporal coordination of an action with a predictable external event, an external rhythm (Repp, 2005).

SMS frequently takes place in social contexts in the sense that other humans produce the sequences with which one's movements need to be synchronized. Many examples can be found in musical settings; people tend to nod their head, clap, or dance in synchrony with music performed live by musicians. When listening to music, people generate temporal expectations based on structural regularities related to the musical beat (a periodic pulse), and they are often compelled to produce movements in synchrony with these regularities (Repp, 2005; Large, 2008). The external events with which actions are temporally coordinated can also be actions themselves, such as when the chaotic applause of an enthusiastic audience after a concert morphs into synchronized clapping (Néda et al., 2000). The act of synchronizing movements with sequences that are produced by other humans is not restricted to musical settings. Another classic example of—involuntary—interpersonal coordination is the tendency for two people walking together to synchronize their walking rhythm with each other (van Ulzen et al., 2008). The above mentioned examples are situations from daily life during which SMS occurs more or less spontaneously. But precise synchronization might also be the explicit goal of extensive practice schedules that are intended to achieve artistic or athletic perfection, as in musical ensembles (Keller and Appel, 2010) or rowing crews (Wing and Woodburn, 1995).

Precise yet flexible SMS requires temporal adaptation (reactive error correction) and anticipation (predictive processes) (see Keller, 2008; Repp and Su, 2013). The mechanisms that support these processes are typically studied separately in SMS research. Here we argue that, to get a better understanding of the nature of SMS, it is fruitful to study adaptive and anticipatory mechanisms within a single framework. The goal of the present paper is therefore two fold:

The first aim is to give an overview of the existing literature on the roles of temporal adaptation and anticipation in SMS. To this end, we provide a sketch of what can be considered to be the state-of-the-art in the field of SMS research, covering its main approaches, including those that employ behavioral experimentation, computational modeling, and the study of brain structures and functional processes that support SMS. This brief review serves to illustrate that, although SMS is a basic and fundamental human skill, its workings are far from simple and are not yet fully understood. In our view, an important gap that needs to be bridged is that between research on adaptive and anticipatory processes in SMS. However, it is also the case that research devoted to understanding each class of process alone has taken divergent paths. In an attempt to make steps toward redressing this divergence, we delineate connections between fields of research that are relevant to the investigation of the role of temporal adaptation and anticipation in SMS but that, to our knowledge, have not been linked before (e.g., tau theory and the concept of “strong anticipation,” see section Strong and weak anticipation).

**Figure 1:**
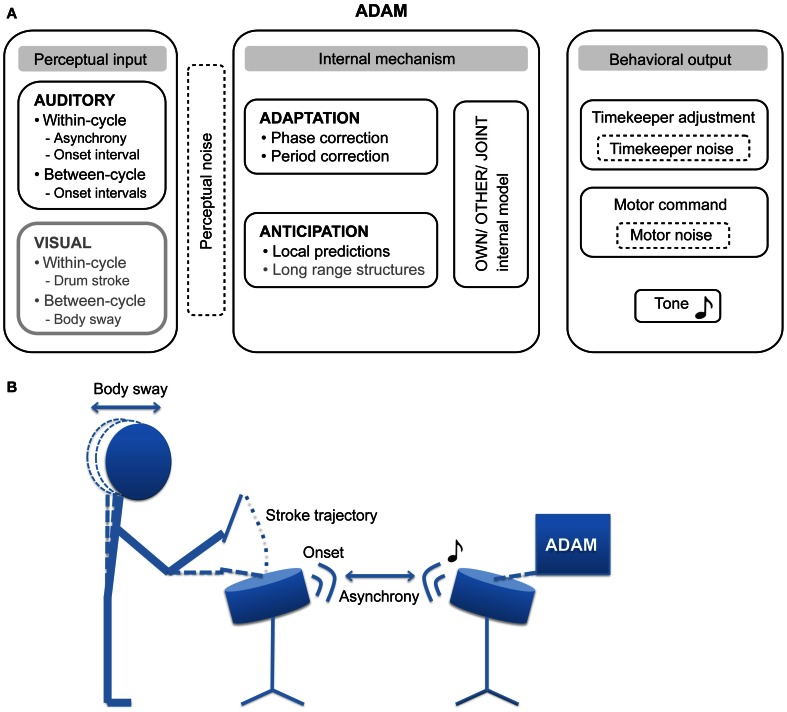
**(A)** The proposed architecture of the ADaptation and Anticipation Model, ADAM. The main components that are illustrated include auditory and visual input, internal mechanisms that support adaptation to and anticipation of this input, and—by communing with internal models of one's own actions, others' actions, and collective joint actions—control behavioral output. In addition, three sources of noise (perceptual, timekeeper, and motor) can affect ADAM's SMS accuracy and precision. Components illustrated in gray have not been implemented in the current version of ADAM, though their relevance to SMS is discussed in this article. **(B)** An example of a bi-directional experimental set up in which the participant and ADAM influence each other in the context of a joint drumming task.

The second aim is to introduce ADAM (Figure 1), an ADaptation and Anticipation Model that is intended to account for both adaptive and anticipatory aspects of SMS. After introducing ADAM, we give a brief overview of novel research paradigms that employ the model in the context of computer simulations and behavioral experiments. These simulations and experiments permit different aspect of SMS—specifically, the effects of, and the link between, adaptation and anticipation mechanisms—to be investigated systematically within a unified theoretical framework.

## Sensorimotor synchronization

SMS is a form of referential behavior in which actions are timed relative to an external event, the referent (Pressing, 1999; Repp, 2005). There is a long tradition of studying SMS in laboratory tasks that require participants to produce simple movements (such as finger taps) in time with events in computer-controlled pacing sequences (e.g., tones). In such paced finger tapping tasks, participants are asked to tap their finger with a specific phase and/or period relation to the timing of an auditory or visual pacing sequence. It is standard practice for participants to be instructed to tap with their index finger in synchrony with the tones produced by a metronome they hear over headphones, while keeping the beat of their taps as stable as possible. Drum strokes may be substituted for finger taps, as shown in the drumming task illustrated in Figure 1B.

A common variable of interest when assessing SMS accuracy is the timing error—the asynchrony—between the occurrence of the action (the drum stick contacting the drum) and the pacing event (see Figure 1B). Although asynchronies can vary in magnitude across experimental conditions and cohorts, and large inter-individual differences are usually evident, the action typically precedes the event, resulting in what has been termed a “negative mean asynchrony” (Aschersleben, 2002). The level of participants' synchronization skill is also reflected in measures of precision, including the variability of asynchronies (i.e., an inverse index of the strength of sensorimotor coupling) and the variability of the intervals between consecutive movements (i.e., an inverse index of stability in tapping tempo) [see (Repp, 2005); Repp and Su (2013) for extensive reviews on SMS].

Although SMS is a widespread and fundamental human skill, individual differences in SMS ability can be observed (e.g., Pecenka and Keller, 2009, 2011; Repp, 2010; Repp and Su, 2013), with certain individuals exhibiting marked impairment in SMS in some contexts (Phillips-Silver et al., 2011). The sources of these individual differences may lie in variations of three broad classes of functional processes that underlie SMS: (1) the perception of timing in rhythmic stimuli, and, specifically a periodic beat, (2) the production of rhythmic movements, and (3) the multisensory integration, or coupling, of perceived rhythms and produced motor rhythms (see Phillips-Silver et al., 2010).

Neuroscientific work on SMS has revealed that these processes are implemented in an extensive network of brain regions, including the primary sensorimotor cortices, premotor cortex, inferior parietal cortex, the supplementary motor area, the cerebellum, and the basal ganglia [e.g., Witt et al., 2008; Coull et al., 2011; see Repp and Su (2013) part 4 for a review on the neuroscience of SMS]. The cerebellar-premotor network seems to be of particular importance for SMS presumably because this network is involved in sensorimotor coordination (Molinari et al., 2007) and audio-motor coupling (Chen et al., 2009). It follows that damage to regions within this network may impair SMS skills, and thus have detrimental effects on the fulfillment of daily activities. To understand the specific sub-components of SMS skill that may be affected, it is helpful to unpack SMS in terms of the sensory modalities that it may involve, the processes that characterize interactions between mutually responsive agents in naturalistic SMS tasks, and the mechanisms related to temporal error correction and prediction in SMS.

### SMS between mutually responsive agents

Studies related to SMS traditionally investigate participants' synchronization with a pacing sequence that is either isochronous or perturbed in more or less systematic ways [see Repp (2005) for review]. The coupling in these cases is unidirectional: the human participant synchronizes with the unresponsive pacing sequence. But in daily life, most of SMS activities involve two mutually responsive agents that are coupled bidirectionally (e.g., interpersonal coordination, musicians playing together). Recently, several paradigms have been introduced to investigate these types of SMS activity.

#### SMS between a human and virtual partner

Following a precedent set by Vorberg (2005), Repp and Keller (2008) examined mutual adaptation during SMS using a paradigm that required interaction with an “adaptive virtual partner.” Specifically, the task entailed a human participant tapping a finger in time with a computer-controlled auditory pacing signal that simulated the potential behavior of a human partner by adapting to the participant's tap timing to varying degrees. This allowed the bidirectional coupling between two interacting agents to be studied under conditions where the behavior of one agent (the virtual partner) was under experimental control. The advantage of this approach is that the effects of parametric variations in adaptation strategy (programmed into the virtual partner) on the behavior of the human participant can be assessed.

Taking a different approach, Kelso et al. (2009) employed a real-time interaction paradigm involving visually mediated coupling between a human and a virtual partner to investigate the dynamics of basic human social coordination. The virtual partner was an avatar of a hand whose movements were driven by a non-linearly coupled component oscillator of the Haken–Kelso–Bunz (HKB) model, a model of basic coordination dynamics. The original HKB model described phase transitions between two hands (Haken et al., 1985). Since then, it has been shown that the HKB equations can be used to describe rhythmic coordination between similar effectors (e.g., fingers) as well as between different effectors (e.g., arm-leg) and even between two individuals (e.g., Kelso, 1995; Schmidt and Richardson, 2008). In the study by Kelso et al. (2009) participants were coupled with the virtual partner via the visual modality. The coupling term for the oscillator used the participant's finger position and velocity to adapt to the participant's performance. Having participants interact with the virtual partner based on the HKB model therefore created an opportunity to investigate reciprocal coordination. Results showed that being reciprocally coordinated with the virtual partner led to different levels of stability and novel behavioral strategies employed by the participants. For example, participants transiently switched between in-phase and anti-phase relations or varied the spatial amplitude of their movements relative to the virtual partner in order to maintain synchronization.

Both of the studies described above (Repp and Keller, 2008; Kelso et al., 2009) combined the use of experiments involving a human participant and a virtual partner with the use of simulations in order to arrive at a better understanding of the observed behavior and its underlying mechanisms. In doing so, these paradigms were successful in identifying new characteristics of SMS behavior.

#### SMS between two humans

Related work that investigated interaction between live humans in dyadic SMS tasks has revealed evidence for mutual temporal adaptation. Konvalinka et al. (2010) explored the ongoing dynamics that result from a coordinated joint tapping task under different coupling conditions created by varying the auditory feedback settings. Participants were asked to maintain a given beat while producing or synchronizing with an auditory signal. The auditory signals were produced either by the participant's own taps, the other person's taps, or the computer metronome. In the metronome conditions, both participants heard only the computer sounds. In the uncoupled condition each participant only heard sounds triggered by his or her own taps, in the unidirectional coupling condition both participants heard taps generated by just one of them, while in the bidirectional coupling condition both participants received the taps generated by the other participant. Results showed that participants were able to synchronize equally well with a human partner that was relatively unpredictable but responsive and with a predictable but unresponsive computer metronome. Furthermore, in the bidirectional coupled condition, the lagged cross-correlations of interpersonal inter-tap intervals showed negative lag-0 and positive lag-1 cross-correlations (e.g., if one participant produced a relatively long interval, the next interval produced by the other individual would be relatively long, suggesting assimilation at the level of inter-onset interval timing). The authors concluded that synchronization between two participants was characterized by continuous adaptation on a millisecond timescale by both individuals.

Taking a different approach, Nowicki et al. (2013) employed a dyadic finger-tapping task in which paired musicians were required to tap in alternation, in synchrony with an auditory pacing signal. Serial dependencies between successive asynchronies produced by alternating individuals' taps relative to the pacing tones revealed evidence for mutual temporal assimilation—a form of behavioral mimicry—when both individuals' taps generated auditory feedback. This result suggested that mutual adaptive timing is characterized more strongly by temporal assimilation than by a compensation processes whereby individuals correct each other's timing errors.

## Adaptation: reactive, error correction mechanisms

SMS, like any human behavior, is characterized by biological noise that leads to variability in movement timing and, therefore, temporal error even when synchronizing with a regular pacing signal. Many instances of SMS, however, involve coordination with signals containing deviations from regularity (e.g., expressive timing in music performance), leading to some degree of uncertainty about event timing and, again, temporal error. Adaptation processes that correct these errors are hence necessary to sustain SMS and to behave with temporal flexibility in the face of this variability. Without these error correction processes, which compensate for timing errors in a reactive fashion, variability would accumulate from movement cycle to movement cycle. This would result in increasingly large asynchronies, phase drift, and the eventual loss of synchronization (Vorberg and Wing, 1996).

### Models of error correction

The modeling of error correction in SMS has been done in myriad ways. Two main approaches can be distinguished: dynamic systems theory and information-processing theory. Dynamic systems models of SMS deal with the relative phase of periodic oscillators, while information processing models posit internal clocks, or timekeepers, that measure or generate discrete time intervals.

Dynamic systems theory assumes that an external rhythmic signal evokes intrinsic neural oscillations that entrain to periodicities in the rhythmic sequence (Large, 2008). The focus within this field is on continuous, non-linear, and within-cycle coupling between these oscillations and the pacing signal. SMS behavior can therefore be modeled with non-linearly coupled oscillators that are described formally in terms of differential equations (e.g., Schöner and Kelso, 1988; Fink et al., 2000; Assisi et al., 2005; Torre and Balasubramaniam, 2009). According to these models, the accuracy and precision of SMS vary as a function of the strength of the phase entrainment of the oscillation to the stimulus sequence, which is defined by a coupling term. To maintain synchrony when the tempo of the stimulus sequence undergoes change, an additional term reflecting period matching is necessary (Large et al., 2002).

The information-processing theory focuses on cycle-to-cycle correction of timing errors and uses linear timekeeper models to model this error correction process. According to timekeeper models, error correction can be described according to linear autoregressive processes (Wing and Kristofferson, 1973; Vorberg and Wing, 1996). These processes produce local dependencies between successive taps: the deviation of the current tap from the mean inter-tap interval and mean asynchrony is proportionally related to the deviation of the inter-tap interval and asynchrony associated with the previous tap plus random noise (Wing and Kristofferson, 1973; Wing and Beek, 2002). The models assume the existence of a timekeeper and different sources of timing variability. The timekeeper functions as a clock that generates pulses, which initiate motor commands (Wing and Kristofferson, 1973). Two additive sources of random timing variability are noise in the timekeeper (related to variability in neural activity) and in the execution of motor commands (due to variable transmission delays in the peripheral motor system). Timing variability can result in large asynchronies and tempo drift. Error correction mechanisms counteract these effects of variability and therefore contribute to maintenance of synchrony with external stimulus sequences (Mates, 1994a,b; Vorberg and Wing, 1996; Vorberg and Schulze, 2002).

It has been argued that the information-processing theory and the dynamic systems theory are closely related, as both can be regarded as variants of a general control equation for referential behavior (Pressing, 1999). However, situations have been documented in which one approach fares better than the other in explaining observed behavioral patterns, as in a recent study that favored a dynamic systems model of synchronized finger tapping with sequences containing gradual tempo changes (Loehr et al., 2011). An alternative to the view that dynamic and information-processing models are essentially equivalent posits that the approaches account for different synchronization processes, and are therefore better suited to explain distinct aspects of SMS (Repp, 2005; Torre and Balasubramaniam, 2009). In the current article, we focus mainly on the information-processing theory because the adaptation module of ADAM is based on work (Repp and Keller, 2008) that took an information-processing approach using autoregressive linear models that account for the behavior in question adequately.

### Phase and period correction

**Figure 2:**
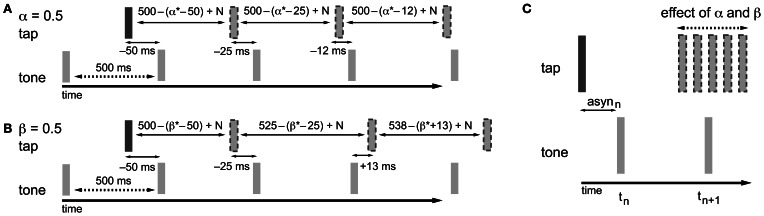
**Adaptation processes based on the asynchrony following the information-processing theory**. In the examples, α and β are both equal to 0.5, which is a value that fits within the typical range of empirical α and β estimates (0.2–0.8). The examples given show how the timing of the next tap is adjusted to compensate for the asynchrony. As a result of the timekeeper setting, the asynchrony (asyn) in combination with error correction mechanisms, and motor and timekeeper noise [*N* (which is set to zero in the present example)] the next tap is shifted in the opposite direction of the asynchrony. **(A)** Phase correction (α = 0.5): half of the asynchrony is corrected. Phase correction is a local adjustment; the setting of the timekeeper (in this example 500 ms) is not affected. **(B)** Period correction (β = 0.5): the correction of the asynchrony has a cumulative effect on the setting of the timekeeper (in this example the base timekeeper is 500 ms), leading to tempo drift. **(C)** In practice, phase and period correction can be both active during SMS. As a result of the combination of both error correction mechanisms, the timing of the next tap is adjusted based on a percentage of the asynchrony.

In the information-processing theory framework, like in the dynamic systems theory, two separate adaptive processes (namely phase and period correction) have been proposed (Mates, 1994a,b; Vorberg and Wing, 1996; Semjen et al., 1998). Both processes independently modify the timing of the next action based on a percentage (α for phase correction; β for period correction) of the asynchrony (Repp and Keller, 2008) (Figure 2) or the difference between the preceding inter-onset interval and the preceding timekeeper interval (Hary and Moore, 1985, 1987). Phase correction is a local adjustment to the interval generated by the internal timekeeper, leaving the period of the timekeeper unaffected (Figure 2A). Phase correction is automatic and does not require conscious registration of the timing error (Repp, 2001a, 2002a), although the gain of implemented phase correction can be manipulated voluntarily to some extent, for example, by suppressing the tendency to react to perturbations (Repp, 2002a; Repp and Keller, 2004). Furthermore, participants can implement phase correction in advance of an expected perturbation to reduce timing errors (Repp and Moseley, 2012). When systematic tempo fluctuations exceed a certain threshold, depending on several parameters like the base tempo (e.g., Takano and Miyake, 2007), phase correction alone is insufficient for maintaining synchronization and the additional process of period correction is necessary. Period correction adjusts the period of the timekeeper that drives the motor activity, and this change to the timekeeper period persists until period correction is applied again (Repp, 2001b) (Figure 2B). Based on simulations, Schulze et al. (2005) proposed that additional control mechanisms function to set the gain of period correction and can thereby determine when it is started and stopped. Period correction is largely under cognitive control, requires attentional resources, and relies on the conscious perception of a tempo change in the pacing sequence (Repp and Keller, 2004).

A number of techniques can be used to estimate the amount of phase correction implemented by humans (see Repp et al., 2012). One technique that has been employed extensively is a perturbation method that involves introducing an abrupt change to a single pacing inter-onset interval in order to examine the so-called “phase correction response” in taps that follow this change (e.g., Repp, 2001a). Recently, Repp et al. (2012) compared the perturbation method with two different methods for estimating phase correction that are based on data from tasks that require participants to synchronize with a regular metronome or an “adaptively timed” pacing signal. In the latter two methods, the estimation of the amount of phase correction implemented by humans is based on analytical techniques that examine the degree of autocorrelation in the time series of asynchronies between finger taps and pacing events. Repp et al. (2012) found that estimates of the amount of phase correction implemented by humans obtained with the regular and “adaptively timed” methods were strongly correlated while estimates obtained with the perturbation method were uncorrelated. According to the authors, these results suggest that keeping in synchrony with a metronome that contains occasional timing perturbations requires different phase correction mechanisms than when synchronizing with a regular metronome or an “adaptively timed” signal (Repp et al., 2012). Estimating the amount of phase and period correction implemented by humans is possible through the use of two-process error correction models that account for short-term phase correction and longer-lasting period correction within a single model (Mates, 1994a,b; Vorberg and Schulze, 2002; Schulze et al., 2005). Repp and Keller (2004) estimated error correction by applying a two-process error correction model (Mates, 1994a,b) to tapping data obtained in a task that required synchronization with a metronome that implemented an abrupt tempo change, after which the participant was required to continue tapping at the new tempo [see Repp and Keller (2004) for a detailed description]. Phase and period correction estimates depend on several aspects of the experimental method (e.g., tempo, task, and analytical technique) but typically vary between 0.2 and 0.8 (Repp and Keller, 2004; Fairhurst et al., 2012; Repp et al., 2012).

### Adaptation mechanisms in the brain

Further evidence that phase and period correction are distinct processes comes from studies using different procedures to investigate brain function [functional *magnetic resonance imaging* (fMRI), electroencephalography (EEG), and *transcranial magnetic stimulation* (TMS)] (see Witt et al., 2008; Repp and Su, 2013). This work has revealed extensive subcortical and cortical networks [spanning the cerebellum, basal ganglia, premotor cortex, (pre-)supplementary motor area, sensorimotor cortex, superior temporal gyrus, and inferior frontal gyrus] that exhibit different patterns of functional connectivity depending on whether error correction is automatic or effortful (Rao et al., 1997; Jäncke et al., 2000; Oullier et al., 2005; Chen et al., 2008; Thaut et al., 2009; Bijsterbosch et al., 2011a,b). An EEG study that specifically targeted the distinction between phase and period correction using source localization placed the former in auditory and secondary somatosensory cortices and the latter in medial frontal cortex, particularly the supplementary motor area (Praamstra et al., 2003).

The brain-based distinction between automatic and effortful error correction has received further support from a recent fMRI study of SMS with virtual partners (Fairhurst et al., 2012). This study, which required participants to synchronize with an adaptive virtual partner that implemented varying degrees of phase correction, highlighted the importance of the hippocampus, precuneus, posterior cingulate, and cuneus cortex for successful synchronization (Fairhurst et al., 2012). Moreover, when the adaptive partner was easier to synchronize with (i.e., when it implemented moderate degrees of phase correction), cortical midline structures were strongly activated. However, when synchronizing with an overly adaptive virtual partner that made the interaction more cognitively challenging, lateral prefrontal areas were recruited to a greater degree. This shift between brain areas suggests a link between action and social processes related to cooperation in the former case and brain areas associated with cognitive control in the latter case, and might indicate an increase in phase correction or the engagement of period correction by the participants (Fairhurst et al., 2012).

## Anticipation: predictive mechanisms

Anticipating the precise onset of stimulus events is important for successful SMS because it allows an individual to get his or her response under way early enough so as to coincide with the event (Schmidt, 1968). To achieve this, the brain has evolved the capacity to extract structural regularities rapidly from ongoing events in the environment, and to use this information as a basis for generating online predictions about the immediate future (e.g., Schubotz, 2007; Friston and Kiebel, 2009). These predictions can coevolve via two routes, one characterized by automatic bottom-up expectancies and the other by top-down processes involving mental imagery (Vuust et al., 2009; Keller, 2012).

Evidence for the involvement of anticipatory mechanisms in SMS comes from several sources. One SMS-related phenomenon that has been attributed to predictive processes is the negative mean asynchrony—indicating that participants' finger taps precede pacing signal tones—that is often observed in simple finger tapping tasks. It has been suggested that the negative mean asynchrony provides evidence that participants anticipate the occurrence of the events in the pacing signal, rather than simply reacting to each successive pacing event, to ensure that (relatively slow) somatosensory feedback from finger taps coincides with (faster) auditory feedback from pacing events [(Aschersleben, 2002); for a review see Repp (2005)]. However, it may also be the case that the negative mean asynchrony reflects the perceptual underestimation of the time interval between the stimulus events (Wohlschläger and Koch, 2000) resulting in a timekeeper setting that is slightly shorter than the pacing interval (Repp and Keller, 2008), which is not necessarily related to anticipation.

Clearer evidence for anticipation mechanisms related to SMS comes from musical activities. When musicians play together, actions need to be coordinated with a high precision but also flexibility to create a coherent piece of ensemble music. Trained ensemble musicians typically show asynchronies in the order of 30–50 ms between tones that, according to the notated score, should be played simultaneously (e.g., Rasch, 1979; Keller and Appel, 2010; Keller, in press). These small asynchronies, indicating a high level of temporal precision, are suggestive of predictive mechanisms related to SMS, as the asynchronies are too small to be the result of purely reactive mechanisms [the fastest reaction times to auditory stimuli are in the order of 100 ms, with average times being around 160 ms (Galton, 1899)] (Keller, 2008).

Furthermore, evidence for anticipation during SMS can be found in studies focusing on the abilities of participants to tap along in synchrony with pacing stimuli (simple tone sequences or musical pieces) that contain gradual tempo changes (Repp, 1999, 2002b; Rankin et al., 2009; Pecenka and Keller, 2011). The relevant dependent variable in these studies is a ratio based on the lag-0 and lag-1 cross-correlation between inter-tap and inter-stimulus intervals. This ratio reflects the degree to which an individual's taps anticipate (“predict”) or follow (“track”) the tempo changes. If an individual tends toward predicting tempo changes (ratio > 1), then the lag 0 cross-correlation coefficient is high relative to the lag 1 cross-correlation coefficient (i.e., the prediction/tracking ratio is greater than 1), because prediction leads to a close match between the current inter-tap and inter-stimulus interval. A tendency to track (ratio < 1), on the other hand, is reflected in higher lag 1 than lag 0 cross-correlations (prediction/tracking ratios less than one) because the current inter-tap interval will most closely match the previous inter-stimulus interval when tracking (Repp, 2002b; Pecenka and Keller, 2011). Prediction and tracking are not mutually exclusive, as an individual can simultaneously engage in both behaviors to some degree.

The tendency to predict tempo changes has been found to differ between individuals in a manner that is positively correlated with musical experience (Pecenka and Keller, 2009). Prediction/tracking tendencies are, furthermore, stable over time and they are able to account for how accurately and precisely an individual synchronizes with computer controlled pacing sequences, as well as how accurately and precisely two individuals synchronize with one another during dyadic finger tapping (Pecenka and Keller, 2011). Studies on prediction during SMS have revealed that prediction can take place at multiple timescales. Local predictions at short timescales (between-cycles) are evident in the observed over- and undershooting that occurs when the tempo alternates between increasing and decreasing in sequences with smooth tempo changes over multiple intervals. Long-range (fractal) scaling of tap timing suggests that global prediction at longer timescales takes place when synchronizing with musical pieces that contained serial correlation (dependencies between the timing of consecutive events) and fractal scaling (long-range correlations affecting non-consecutive events) (Rankin et al., 2009).

### Anticipation and internal models

It has been claimed that anticipatory mechanisms that subserve SMS with tempo changing sequences are grounded in online action simulations and internal models (Keller, 2008, 2012). Action simulation occurs when sensorimotor brain processes that resemble those associated with executing an action are engaged in a manner that does not directly produce overt movement (Decety and Grezes, 2006; Rizzolatti and Sinigaglia, 2010). The process of action simulation is supported by internal models that represent the sensorimotor transformations that mediate intentions, motor commands, and behavioral effects. Internal models can run independently of action execution, and they can therefore be used to generate predictions about the effects of the intention to perform a particular act, and of a specific movement (Wolpert and Kawato, 1998). Two types of internal model have been distinguished: forward and inverse models. Forward models represent the causal relationship between the input and output of the action control system. They predict the effect that a particular motor command will have upon the body and the dynamic environment, given the current state of the action control system. Inverse models, on the other hand, provide the motor command that is necessary to produce a desired change in state of the body and the environment. By providing motor commands, inverse models serve as controllers for intentional action (Wolpert and Kawato, 1998).

Forward and inverse models are tightly coupled and together facilitate efficient motor control by allowing potential movement errors to be corrected in advance. Internal models can be employed to make predictions about others' actions, including the timing of these actions (e.g., Knoblich and Jordan, 2003; Wolpert et al., 2003; Blakemore and Frith, 2005; Wilson and Knoblich, 2005; Keller, 2008). Utilizing these predictions of future events during the planning of one's own actions is important for successful interpersonal coordination (Knoblich and Jordan, 2003; Konvalinka et al., 2010; Vesper et al., 2011). The predictive abilities of the motor system can extend from actions to external events more generally, which allows for the prediction of spatiotemporal properties even of event sequences that humans are not capable of producing themselves (e.g., when a wave will hit the coast) (Schubotz, 2007). Internal models thus provide an effective mechanism for anticipating future events, and for controlling behavior accordingly, which is a crucial aspect of successful SMS.

### Strong and weak anticipation

A relatively recent development related to anticipation is the distinction between “strong anticipation” and “weak anticipation.” Weak anticipation refers to anticipation based on a model of the environment (akin to an internal model). Strong anticipation is based on anticipation of the system itself and relies on systemic lawfulness, a dynamic process in which behavior adapts itself to the global statistical structure of the environment (Stepp and Turvey, 2010). Strong anticipation can therefore occur without any reference to an internal model (Dubois, 2003). In the former (weak) case, anticipation involves prediction and expectation, whereas in the latter (strong) case, anticipation arises from lawful regularities between a system and its environment, rather than from a process of action planning that takes future states of the environment into account (Dubois, 2003; Stepp and Turvey, 2010). Strong anticipation is thus not about solving a model of the predicted future but instead about keeping specific relationships between components of the to-be-performed task stable and, by doing so, the future states of the components will emerge without the need for an active process of prediction.

An apt example of strong anticipation in music can be found in work on general tau theory (although strong anticipation is not explicitly mentioned in this work) (Lee, 1998; Lee and Schögler, 2009). The general tau theory assumes that purposeful movements involve closing “gaps” between the current state of the body and a goal state. For example, successful violin playing entails controlling the closure of the gap between the initial position of the bow and the end position, to produce the desired tone. According to the general tau theory, the only variable necessary to guide the gap closure is the time-to-closure of the gap at the current closure rate (tau). An intrinsic tau, necessary to close the gap, is computed in the brain, and while playing the violinist tries to maintain a constant relation between this intrinsic tau and the actual tau of the gap, which changes during the movement (Lee and Schögler, 2009). By keeping the ratio constant, it is not necessary to use a model to predict the appropriate movement; the movement emerges as the gap closes.

Weak and strong anticipation may play complementary roles in SMS. Weak anticipation has been linked to event-based timing, as conceptualized by the information processing theory, while strong anticipation is more closely aligned with emergent timing, which is a key feature of the dynamical systems theory (Torre and Balasubramaniam, 2009; Marmelat and Delignières, 2012). Although the exact role of the processes and how the two interact with each other is still unclear, weak anticipation may subserve local timing at short time scales while strong anticipation may be relevant to global timing at long time scales. Thus, weak anticipation may entail local predictions generated via action simulation and internal models, while strong anticipation arises naturally as a consequence of the presence of long-range correlations in environmental event sequences and behavior performed in synchrony with these event sequences (Stephen et al., 2008; Marmelat and Delignières, 2012).

### Anticipation in the brain

The distinction between types of anticipation that differ in terms of timescale and whether they are generated in a top-down or bottom-up fashion is reflected in different brain networks, which nevertheless interact with one another. The extent of these networks is large, prompting Bubic et al. (2010) to point out that the “predictive brain” is in fact the whole brain. However, it is still possible to paint a picture in broad brushstrokes where higher-level areas (e.g., premotor and lateral, medial and prefrontal regions) formulate expectations that are communicated to sensory, lower-level areas (Bubic et al., 2010). These top-down mechanisms could then interact with predictions that are generated automatically in a bottom-up manner in sensory areas such as the auditory cortex (see Bendixen et al., 2012).

A recent fMRI-study by Pecenka et al. (submitted), which investigated synchronization with tempo changing sequences, highlights the extent of the brain network that is involved in generating predictions at multiple levels during SMS. This study identified a large-scale network of areas—including superior temporal gyrus, medial orbitofrontal cortex, midcingulate cortex, posterior cingulate gyrus, and cerebellum—in which activation was related to behavioral measures of the degree to which tempo changes were anticipated. This study, taken together with other work on temporal prediction (Schubotz, 2007; Leaver et al., 2009), suggests that the mixture of processes related to action simulation and expectancy generation during SMS is orchestrated by a network of cortical areas (including prefrontal cortex, inferior frontal gyrus, premotor cortex, superior/middle temporal gyrus, and sensorimotor cortex) that communes with internal models in cerebellum (see Wolpert et al., 1998; Fleischer, 2007; Ito, 2008; Coull et al., 2011).

## ADAM: the adaptation and anticipation model

The literature reviewed in the foregoing sections of this article suggests that adaptation and anticipation mechanisms are involved when synchronizing actions to external events. To get a more complete idea about what the role of both mechanisms is, how they are linked, and how they influence each other, it is desirable to consider both mechanisms within a unified framework. In the following paragraphs we introduce ADAM (Figure 1), an ADaptiation and Anticipation Model as an appropriate next step in the process of disentangling the reactive and proactive processes that underpin SMS.

Our goal in creating ADAM was to provide a novel tool that can be employed to explore different sensory modalities (e.g., auditory and visual input) and timescales (within-cycle, between-cycles, and long range structures) in SMS. Furthermore, we propose that ADAM can assist in evaluating the degree of motor impairment and can be used in guiding patients through motor rehabilitation. The current article describes how ADAM handles auditory input, taking within- and between-cycle information into account when computing its behavioral output via timekeeper adjustment and the issuing of “motor” commands.

ADAM is an ADaptation and Anticipation Model that combines the adaptive model used by Repp and Keller (2008) with an anticipation process instantiated as an internal model. Combining adaptation and anticipation within one framework holds the potential to shed light on the relation between these mechanisms by allowing a direct comparison of the effect of adaptive timing and anticipatory processes on SMS. To provide an intuitive description of ADAM, in the current article, a drumming paradigm in which ADAM produces the stimulus sequence by means of percussion sounds and the human participant strikes a drum is used to describe the model. Obviously these sounds can be substituted by any discrete event and drum strokes are simply a convenient, exemplary action. For the sake of consistency with the large body of research on finger tapping with pacing sequences comprised of tones, we refer here to percussion sounds as “tones” and drum strikes as “taps.”

### Adaptation with ADAM

**Figure 3:**
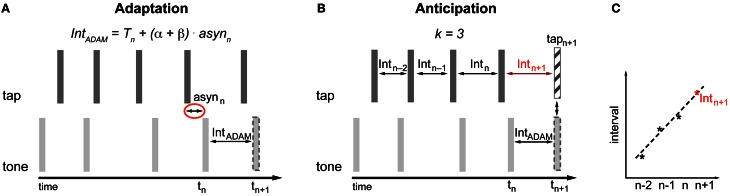
**Implementation of adaptation (A) and anticipation (B,C) in ADAM**. asyn, asynchrony; Int, interval; T, Timekeeper period. **(A)** The time of occurrence of the next tone is determined based on the asynchrony and the settings of error correction parameters α and β (Equation 1). The setting of β affects the current timekeeper period (Equation 2). The interval produced by ADAM between *t*_*n*_ and *t*_*n*+1_ is equal to *T*_*n*_ + (α + β) × asyn_*n*_. **(B,C)** A curve-fitting process that is applied to the preceding intervals predicts the participant's next tap time (Equations 3 and 4). The timing of upcoming tone or the next interval produced by ADAM can be set to enable ADAM's next tone to coincide with the predicted next tap of the participant.

The adaptive module is conceived in the spirit of the information processing approach. Therefore the occurrence of the next tone (*t*_*n*+1_) produced by ADAM is based on a timekeeper (*T*) with additional phase (α) and period (β) correction linked to the asynchrony (asyn) between the tone produced by ADAM and the tap produced by the participant (Figure 3A). The adaptive module uses a two-process error correction model (Repp and Keller, 2008) that can be described by the following equations^1^:

(1) $$t_{n+1}=t_{n}+T_{n}+\left(\alpha +\beta \right)\times {\text{asyn}}_{n}$$

(2) $$T_{n+1}=T_{n}+\beta \times {\text{asyn}}_{n}$$

The most recent asynchrony (asyn_*n*_) is multiplied by the sum of the phase (α) and period (β) correction parameters and the result is added to the current timekeeper period (*T*_*n*_) in order to obtain the current tone inter-onset interval (*T*_*n*_ + (α + β) × asyn_*n*_). This current tone inter-onset interval is added to the onset time of the current tone (*t*_*n*_) to calculate the time of occurrence of the next tone (*t*_*n*+1_) (Equation 1). Period correction is a lasting change of the timekeeper setting. To accomplish this, the next timekeeper period (*T*_*n*+1_) is given by the last asynchrony (asyn_*n*_) multiplied by the period correction parameter (β) added to the current timekeeper (*T*_*n*_) (Equation 2).

The phase (α) and period (β) correction parameter can be set separately. The settings of α and β cause ADAM to implement phase or period correction, or a combination of both error correction mechanisms (Equations 1 and 2). Repp and Keller (2008) used a similar adaptive virtual partner that varied α between −1 and 1 and β between 0 and 1. Setting both parameters to 0 results in a conventional non-responding metronome, while an α less than 0 leads to negative phase correction (onset of the tone shifts in opposite direction to the asynchrony), which makes SMS difficult for the participant. Optimal phase correction, operationally defined as the α value that minimizes the variability of asynchronies, is achieved with an α between 0.3 and 0.5, both for the adaptive pacing signal and humans (Repp and Keller, 2008; Fairhurst et al., 2012). A phase correction parameter of 1 would be perfect phase correction, an α of 2 would imply over-correction and settings greater than 2 result in instability (Repp and Keller, 2008).

Repp and Keller (2008) showed that participants are capable of synchronizing with different types of adaptive virtual partners that implement varying degrees of (positive and negative) phase and/or period correction. Strategies used by the participant to maintain synchrony with the virtual partner were determined with the help of computer simulations. The simulations aimed to find error correction settings for the human participants that showed the best fit with empirical data across the parameter settings employed by the virtual partner. Results showed that strategies differed as a function of the settings of the adaptive virtual partner. For example, participants implemented a fixed gain of phase correction as long as the adaptive partner was cooperative (i.e., the partner implemented a small-to-modest amount of positive phase correction), while the error correction strategy of the participants changed when participants were dealing with an uncooperative adaptive partner (i.e., the partner implemented negative phase correction). Furthermore it turned out to be important that participants assumed responsibility for maintaining the correct tempo when the virtual partner implemented period correction and was therefore liable to drift [see Repp and Keller (2008) for additional findings]. With the adaptation module ADAM, we are able to replicate the computer simulations and patterns of effects.

### Anticipation with ADAM

The anticipatory module in ADAM is based on a temporal extrapolation process that generates a prediction about the timing of the participant's next tap based on the most recent series of inter-tap intervals that ADAM receives as input (Figures 3B,C). This temporal extrapolation process works by extending systematic patterns of tempo changes in such a way that a decelerating sequence with inter-tap intervals that increase in duration will result in a prediction that the next tap will occur after an even longer interval, and vice versa for tempo accelerations. The timing of the participant's next tap is determined via curve fitting: An over-determined linear system based on at least three inter-tap intervals (*k* ≥ 3) is created and solved to find the straight line that fits best to the intervals. The line that fits best is defined as the one that minimizes the sum of the squared errors between the line itself and the interval data^2^. Extrapolating from this best-fitting function, the upcoming inter-tap interval of the participant is predicted (Int_*n*+1_) (Figure 3C). Equation 3 is used to determine this predicted interval. The predicted time of the next tap (tap_*n*+1_) is based on Equation 4.

(3) $${\text{Int}}_{n+1}=a+b\times \left(n+1\right)$$

(4) $${\text{tap}}_{n+1}={\text{tap}}_{n}+{\text{Int}}_{n+1}$$

In Equation 3, *a* represents the intercept and *b* stands for the slope of the best fitting line. Both parameters *a* and *b* depend on the number of intervals (*k*) used to determine the best-fitting straight line. Based on this predicted next tap it can be determined when the next tone produced by ADAM should occur or what the next interval of ADAM should be (Int_ADAM_) in order for ADAM's next tone (*t*_*n*+1_) to coincide with the predicted next tap of the participant (tap_*n*+1_) (Figure 3B). The anticipatory module of ADAM thus constitutes an over-determined system in which the number of intervals (*k*) used to create and solve the linear system can vary, but at least three intervals are used (a minimum of two intervals is necessary to find a straight line). An over-determined system is useful when dealing with noisy data—such as those that arise, for example, due to variability in human sensorimotor systems—because the error resulting from the noise is averaged out when fitting the line to multiple intervals.

The above implementation of anticipation in ADAM leads to patterns of inter-onset intervals that are classified as the behavior of a “predictor” as the lag 0 cross-correlation between the inter-tap and inter-stimulus intervals is higher than the lag 1 cross-correlation, and therefore the prediction/tracking ratio will be bigger than 1 (Repp, 2002b; Pecenka and Keller, 2011). Tracking behavior can be produced by introducing into ADAM the tendency to mimic the previous inter-tap interval (cf. Konvalinka et al., 2010).

### Linking adaptation and anticipation mechanisms in ADAM

**Figure 4:**
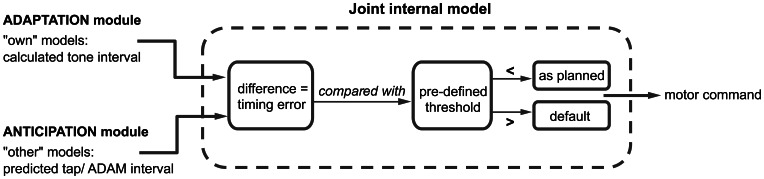
**A schematic overview of the joint internal model, where the adaptation and anticipation module interact via “own” and “other” internal models**. The difference between the outputs of the adaptation and anticipation module of ADAM is compared to a predefined threshold. Depending on this comparison, the motor command is either executed as planned or in accordance with a default setting. A detailed description of this process can be found in the text.

One of our goals when developing ADAM was to shed light on the link between adaptation and anticipation. We hypothesize that a combination of paired internal models used to simulate one's own and others' actions plays a role in this link. Following seminal work by Wolpert et al. (2003), a number of approaches have proposed that such paired forward and inverse models are employed during social interaction (e.g., Pacherie, 2008, 2012). In ADAM, separate classes of forward and inverse models are harnessed to simulate ADAM's own actions and the human participant's actions slightly in advance of their production (Figure 4). The coupling of “own” and “other” internal models facilitates fluent SMS by allowing potential errors in timing to be anticipated and corrected before they occur (Wolpert et al., 2003; Keller, 2008).

ADAM simulates its next action with an “own” inverse model that receives the output of the adaptive module (i.e., the next planned inter-tone interval) and, based on this, selects an appropriate motor command. An “own” forward model then generates a prediction of the timing of the next tone that would result if the motor command were to be carried out. Independently of the operation of these “own” internal models, the anticipatory module of ADAM runs an “other” forward model, which generates a prediction of the participant's next tap-interval and therefore a predicted tap time. In situations characterized by complex but systematic patterns of tempo change, as in expressively performed music, ADAM can be equipped with a template descripting the tempo changes that functions as an inverse model of the other's actions.

The predicted tap-interval of the participant or planned interval of ADAM (based on the “other” model) is compared with the predicted tone-interval (from the “own” model) in a “joint” internal model (see Figure 1). This joint model is where adaptation and anticipation mechanisms interact in ADAM (Figure 4). It essentially simulates the timing error that would arise as a result of the current parameter settings related to reactive error correction and predictive temporal extrapolation processes in ADAM's adaptation and anticipation modules. If the error falls within a pre-defined tolerance region [e.g., a threshold that is based on whether the difference is perceivable or not (Repp, 2001b)], then the motor command is issued and ADAM produces a tone. If not, then ADAM refers to the default mechanism, which is either the interval computed via the adaptation or anticipation module, to set the motor command to produce the next tone. ADAM's complete architecture (Figure 1) includes multiple, hierarchically nested internal models (cf. Pacherie, 2008) that can simulate processes unfolding at different timescales (within- and between-cycle) in different modalities (auditory and visual, see section Extensions of ADAM). Combining adaptation and anticipation mechanisms at multiple levels in ADAM's action control hierarchy engenders SMS that is accurate, precise, and flexible (in the sense that tempo changes can be negotiated).

### Simulations and experiments using ADAM

The purpose of developing ADAM was twofold. The first goal was to provide a platform on which the multiple mechanisms and processes involved in SMS can be systematically explored in computer simulations and behavioral experiments. Under both investigative methods, the parameter settings for the adaptive and anticipatory components of ADAM can be varied in order to test hypotheses about the role of individual components, and the interaction of multiple components, on the accuracy and precision of SMS. Specific questions that we have considered in simulations include (1) the conditions under which adaptive phase correction (α) and period correction (β) processes are necessary and sufficient for stable SMS, (2) the way in which phase correction and period correction are combined (e.g., under-additive, additive, or over-additive), (3) the effects of the type of function (e.g., linear, 2nd order polynomial) and the number of inter-onset intervals used by the anticipation module of ADAM to generate temporal predictions, (4) the relationship between temporal adaption and anticipation (as described in section Linking adaptation and anticipation mechanisms in ADAM), and (5) how varying levels of perceptual, timekeeper, or motor noise affect the optimal settings for parameters governing temporal adaptation and anticipation. The results of simulations addressing these issues have been used to inform the process of designing experiments to test how the effects observed in computer simulations generalize to situations that involve the interaction between ADAM and live human partners. The match between the results of the simulations and the behavior of ADAM with human participants can be used to improve the model in terms of optimizing the goodness of fit.

Several different real-time experimental setups (finger tapping or drumming tasks; see Figure 1B) are possible in which participants and ADAM interact with each other through different coupling regimens (unidirectional vs. bidirectional). These setups allow us to explore social aspects of SMS between two responsive agents. In addition to interrogating ADAM's behavior, this approach lends itself to the investigation of how participants respond to pacing signals associated with different types of interaction partner that ADAM can provide. Questions of interest include how human participants respond (in terms of objective behavior and subjective judgments) to ADAM when it is more or less adaptive or anticipatory. During such experiments, ADAM's parameter settings are known, and therefore the controlled variation of these parameters allows causal connections between adaptive and anticipatory processes and behavioral outcomes to be established.

The second goal in developing ADAM relates to the assessment and rehabilitation of disorders that affect rhythmic movement timing (e.g., Parkinson's disease and stroke-related lesions to areas such as the cerebellum and basal ganglia). It is envisaged that assessment can be carried out using a strategy that combines behavioral experiments and computer simulations. This strategy will allow deficits in specific mechanisms (phase correction, period correction, and prediction at short- or long-time scales) and modalities (auditory and visual; see below) to be identified and linked to lesions identified in structural brain images. Information about specific mechanisms that cause impairment to rhythmic movement timing can then be used in targeted interventions during motor rehabilitation.

### Extensions of ADAM

We envision three future extensions of ADAM: (1) ADAM could make use of visual information from human participants; (2) ADAM could provide visual information to participants; (3) a version of ADAM based on the principles of dynamic systems theory could be created.

Although it is often reported that movements can be synchronized more accurately based on auditory information than with other stimuli, SMS is possible with a variety of stimuli in different sensory modalities (e.g., auditory, visual, tactile) (Repp and Penel, 2004; Hove and Keller, 2010; Hove et al., 2013). Adding spatial variation to the visual stimuli with which participants are required to synchronize—for example, by means of apparent or biological motion—significantly improves participants' synchronization abilities (Hove et al., 2010, 2013), sometimes even leading to performance that is similar to synchronization with an auditory metronome (Hove et al., 2012). To address this aspect of SMS, it would be useful to provide ADAM with visual information from the movements of the participants.

This new component of ADAM could deal with within-cycle (the movement trajectory of a drum stroke or finger tap) and between-cycle (e.g., body-sway) information (Figure 1). SMS studies involving finger movements have demonstrated that features of the produced movement trajectories affect timing accuracy (Balasubramaniam et al., 2004; Balasubramaniam, 2006; Elliott et al., 2009; Hove and Keller, 2010), and this information is presumably available to an individual who intends to synchronize with the observed movements. Furthermore, studies of body movements during music performance have shown that head motion and body sway play a role in regulating performance timing and achieving interpersonal coordination in ensembles (Davidson, 2009; Keller and Appel, 2010).

We propose that combining this visual module with ADAM's auditory module would yield benefits deriving from the fact that information from different modalities play complementary roles during SMS. Consider for example a dyadic drumming task where two individuals synchronize their drum strokes under a regime where they start at a moderate tempo, then gradually accelerate to a fast tempo, and finally decelerate through the initial moderate tempo down to a slow tempo. Each drum stroke—or movement cycle—includes (1) auditory information in the form of a discrete sound with a sharp onset when the drumstick impacts upon the drum, and (2) visual information about the trajectory of the drumstick and the drummer's body movements (Figure 1B). Auditory information (i.e., the onset time of the drum sound) is only available at one time point within a movement cycle, and each sound alone is not informative about how the next movement cycle should be timed. However, sounds associated with successive drum strokes provide between-cycle information—sequences of inter-onset intervals—that can be used to guide movement timing from cycle to cycle. Drumstick and body movement trajectories, on the other hand, are potentially informative about within-cycle and between-cycle timing, respectively. Specifically, the velocity and acceleration of a drum stroke during its descent provides information about the time point of the strike, while body movements—such as head motion and body sway—are informative about timing at longer timescales spanning multiple cycles.

The foregoing suggests that auditory and visual information may assist with different aspects of SMS in the context of challenging coordination tasks, such as those that involve systematic tempo changes. Another way in which information from several sensory modalities may assist SMS is through a multisensory integration process that takes into account the sensory and temporal reliability of events (Elliott et al., 2010). Thus, when multiple information streams are available and the temporal discrepancy between them is small, the combination of information streams in different modalities (e.g., auditory, visual, and tactile timing cues) leads to optimal cue integration and hence more accurate synchronization (Elliott et al., 2010; Wing et al., 2010).

The second proposed extension of ADAM involves using the visual module to drive multimodal displays of virtual synchronization partners that comprise dynamic visual representations of human body segments that move in time with music according to biological kinematic principles. The rationale behind using multimodal displays is that they exploit the benefits of auditory-motor coupling (Zatorre et al., 2007) as well as the tendency for visual depictions of biological motion to induce movement tendencies in an observer (Saygin et al., 2004; Press, 2011). Thus, combined auditory information and continuous biological motion in a virtual synchronization partner based on ADAM should provide a more potent means of driving the participant's movements than either modality alone.

In the context of motor rehabilitation, such multimodal virtual synchronization partners could illustrate the movements that should be synchronized with the music, and they could accompany the patient in executing these movements. Importantly, as noted above, the virtual partner would receive input concerning the patient's behavior via auditory and visual modules of ADAM. It is hypothesized that, by anticipating and adapting to the patient's movement timing and kinematics to varying degrees, the virtual partner would be effective at encouraging as accurate and graceful movement as possible given the individual patient's specific impairment. Furthermore, the parameter settings of the virtual synchronization partner could be adjusted incrementally, leading to different levels of responsiveness and variability that affect the predictability and perceived cooperativity of the partner (Vesper et al., 2011; Fairhurst et al., 2012). These different settings could be used to challenge the patient at later stages of rehabilitation, in order to simulate challenges that arise in complex dynamics environments encountered in daily life.

Finally, we believe that it would be fruitful to develop a version of ADAM based on the principles of dynamic systems theory, or a hybrid version in which both the information-processing theory and the dynamic systems theory are combined. This extension of ADAM could potentially inform the ongoing debate about the validity of the information-processing and dynamical systems theory in relation to SMS. Given that the focus of dynamic systems theory is on continuous, non-linear, and within-cycle coupling (Large, 2008), the envisaged visual module of ADAM that deals with continuous within-cycle information, like drum stroke trajectories (Figure 1), seems especially amenable to the dynamic approach. A virtual partner paradigm similar to that used by Kelso et al. (2009) could serve as a starting point for such an endeavor. Furthermore, the auditory module of ADAM could also be instantiated in a dynamical framework. For instance, work on non-linearly coupled oscillators, described formally in terms of differential equations (e.g., Schöner and Kelso, 1988; Torre and Balasubramaniam, 2009; Loehr et al., 2011), and period matching (Large et al., 2002) provide clear guidance with regard to the steps that could be taken toward a dynamic version of the adaptive module of ADAM. The anticipatory module presents a greater challenge, and it would be worthwhile to evaluate the degree to which the concept of strong anticipation can deal with predictive processes that characterize SMS in challenging contexts that involve tempo changes.

## Conclusion

Adaptation (reactive) and anticipatory (predictive) mechanisms are important for precise yet flexible SMS with externally controlled sequential events. To investigate the role of temporal adaptation and anticipation in SMS, and the link between both classes of mechanism, we introduced ADAM: an ADaptation and Anticipation Model. ADAM combines adaptive, error correction processes with an anticipatory, predictive temporal extrapolation process inspired by the computational neuroscience concept of internal models. ADAM provides a unified framework in which simulations can be combined with experimental manipulations, and therefore constitutes a promising tool for exploring adaptation and anticipation in SMS. In a next step, ADAM could be extended in several ways (e.g., equipped to deal with between-cycle information in the visual modality) to work toward a better understanding of the different aspects of SMS that arise in everyday life, where coordination takes place via multiple modalities and at multiple time scales. ADAM is expected to prove beneficial in advancing our theoretical understanding of basic mechanisms that allow healthy individuals to coordinate their actions with events in the dynamic environment, as well as in the clinical assessment and rehabilitation of individuals with deficits that cause them to struggle with such coordination.

### Conflict of interest statement

The authors declare that the research was conducted in the absence of any commercial or financial relationships that could be construed as a potential conflict of interest.

## Footnotes

^1^Please note the difference in sign of the (positive) error correction terms in Equation 1 compared to the examples in Figure 2 (where they are negative). This difference arises due to the fact that error correction in Figure 2 is specified from the perspective of a human participant, whereas in Equation 1 it is specified from ADAM's perspective.

^2^Following the method of least squares, the line of the form Int = *a* + *b* × *x* has the smallest sum of squared errors if

$$a\times k+b\times \sum_{i\;=\;1}^{k}x_{n-k+i}=\sum_{i\;=\;1}^{k}{\text{Int}}_{n-k+i}$$

and

$$a\times \sum_{i\;=\;1}^{k}x_{n-k+i}+b\times \sum_{i\;=\;1}^{k}{\left(x_{n-k+i}\right)}^{2}=\sum_{i\;=\;1}^{k}\left(x_{n-k+i}\times {\text{Int}}_{n-k+i}\right).$$

The smallest sum of squared errors is obtained if

$$b=\frac{k\times \sum_{i\;=\;1}^{k}x_{i}\times \text{Int}-\;\sum_{i\;=\;1}^{k}x_{i}\times \sum_{i\;=\;1}^{k}\text{Int}}{k\times \;\sum_{i\;=\;1}^{k}x_{i}^{2}-\sum_{i\;=\;1}^{k}x_{i}\times \sum_{i\;=\;1}^{k}x_{i}}$$

and

$$a=\frac{1}{k}\times \sum_{i\;=\;1}^{k}{\text{Int}}_{i}-\frac{1}{k}\times b\times \sum_{i\;=\;1}^{k}x_{i}.$$
